# Unifying mortality forecasting model: an investigation of the COM–Poisson distribution in the GAS model for improved projections

**DOI:** 10.1007/s10985-024-09634-x

**Published:** 2024-09-13

**Authors:** Suryo Adi Rakhmawan, Tahir Mahmood, Nasir Abbas, Muhammad Riaz

**Affiliations:** 1 Department of Social Statistics, BPS-Statistics Indonesia, Jakarta, 10710 Indonesia; 2 School of Computing, Engineering and Physical Sciences, University of the West of Scotland, Paisley, PA12BE UK; 3 Department Mathematics, College of Computing and Mathematics, King Fahd University of Petroleum and Minerals, Dhahran, Saudi Arabia

**Keywords:** COM–Poisson, Count models, Forecasting, GAS model, Time-to-event, 62M10, 62P10, 62N05

## Abstract

Forecasting mortality rates is crucial for evaluating life insurance company solvency, especially amid disruptions caused by phenomena like COVID-19. The Lee–Carter model is commonly employed in mortality modelling; however, extensions that can encompass count data with diverse distributions, such as the Generalized Autoregressive Score (GAS) model utilizing the COM–Poisson distribution, exhibit potential for enhancing time-to-event forecasting accuracy. Using mortality data from 29 countries, this research evaluates various distributions and determines that the COM–Poisson model surpasses the Poisson, binomial, and negative binomial distributions in forecasting mortality rates. The one-step forecasting capability of the GAS model offers distinct advantages, while the COM–Poisson distribution demonstrates enhanced flexibility and versatility by accommodating various distributions, including Poisson and negative binomial. Ultimately, the study determines that the COM–Poisson GAS model is an effective instrument for examining time series data on mortality rates, particularly when facing time-varying parameters and non-conventional data distributions.

## Introduction

Forecasting mortality is crucial in various aspects of life, particularly with the emergence of COVID-19, which has disrupted various societal arrangements (Böhnstedt et al. 2021). The prediction of mortality has become even more relevant and critical. Specifically, mortality forecasting is of utmost importance in evaluating of life insurance company solvency. Forecasts related to mortality are pivotal for determining future obligations to policyholders and gauging the necessary of risk-based capital levels. Given the trend of decreasing mortality rates in numerous developed countries over the years, there is a pressing need for statistical models that can reliably project growth in life expectancy. The Lee-Carter model (Lee and Carter 1992), stands out as a prominent model for predicting mortality trends.

The Lee–Carter model represents the log time series of mortality rates for each age group by using a distinct age intercept and a shared trend that is multiplied by a particular age coefficient. In other words, two parameters are considered in the construction of the Lee–Carter model, namely age-related and time-related parameters. This model utilizes Singular Value Decomposition (SVD) in conjunction with Ordinary Least Squares (OLS) to extract patterns and provide a comprehensive view of the prevailing trend. Additionally, the model incorporates the Auto Regressive Integrated Moving Average (ARIMA) model to extrapolate the general trend, either considering the observed year movement or as a general trend. Consequently, it enables the prediction of mortality rates for any age group.

Various extensions of the Lee–Carter model have been proposed with the dynamic evolution of mortality phenomena. Brouhns et al. (2002) introduces enhancements to the Lee–Carter methodology by tackling the primary limitation of OLS when integrated with SVD, particularly the presumption of consistent errors. They modify the Lee–Carter framework by postulating that death counts adhere to a Poisson distribution (naturally displaying heteroscedasticity). The parameters for this are ascertained using a recursive process tailored for a log-linear construct with dual linear components. Additionally, they employ the ARIMA model for mortality rate predictions. Renshaw and Haberman (2006) also postulate a Poisson distribution for death counts and embed cohort effects within the Lee–Carter approach. The Poisson distribution has also been the focus of many studies in the development of mortality models because it is a natural choice for modeling the death number and provides a formal statistical framework as in the study by Brouhns et al. (2002) and Li (2013) which also extends the Lee–Carter model. In contrast, works by Cossette et al. (2007) and Renshaw and Haberman (2008) delve into the binomial interpretation of the Lee–Carter framework. Delwarde et al. (2007) address the evident over-dispersion in mortality data by suggesting that death counts conform to a negative binomial distribution, thereby refining the Lee–Carter methodology. Lastly, Chen et al. (2017) introduce a dynamic multi-population mortality framework rooted in a two-factor copula, with temporal parameters adjusted via the Generalized Autoregressive Score (GAS) updating technique. The extension of the GAS model under the Bayesian setup is recently provided by Hilton et al. (2019).

The GAS methodology saw advancements by Neves et al. (2017), who incorporated five distinct distributions into the Lee–Carter model under the GAS method. Their study introduced five probabilistic models (Poisson, binomial, negative binomial, Gaussian, and beta), all anchored in the GAS structure, to deduce the Lee–Carter parameters and project mortality rates through a unified single-step method. These frameworks were tested on male mortality rate time series data from the United States, Sweden, Japan, and England. The research utilized quantile residuals for diagnostic evaluations, employed the AIC for model choice, and juxtaposed the predictive accuracy of these models using the Diebold-Mariano assessment. The investigation’s outcome suggests that the negative binomial distribution of the Lee–Carter model within the GAS structure stands out as the most apt for predicting mortality.

Similar findings, where the negative binomial distribution performs better in constructing the Lee–Carter mortality model, are also demonstrated in a study conducted by Azman and Pathmanathan (2022). The researchers analyzed the Poisson, Negative binomial, and Binomial probability distributions within the context of the Generalized Linear Model (GLM) structure associated with the Lee–Carter model. These models were utilized on mortality data from a selection of 10 countries. To evaluate the fit of these models, deviance statistics were used, and standardized residuals were graphically represented against their corresponding fitted values.

However, considering the current dynamics and uncertainties, the choice of distribution becomes crucial, necessitating the adoption of a more general distribution capable of capturing distribution phenomena or even determining multiple distributions simultaneously. The study by Li et al. (2022) proposes the use of Tweedie distributions, building on previous work Jørgensen and Paes De Souza (1994), Taylor (2007), and Peters et al. (2009) to model mortality developing a double modeling framework that combines mean and dispersion models using a family of Tweedie distributions. The results show that this approach can improve mortality modeling performance and provide more accurate risk estimates in the context of long-term risk assessment. The generalizability of models or distributions that can specify multiple distributions simultaneously for mortality cases makes a great opportunity for solutions to the current challenges of mortality dynamics. The COM–Poisson is a distribution that directly captures three distributions: Poisson, Negative binomial, and Bernoulli (Shmueli et al. 2005). This research addresses this hypothesis by developing the application of the GAS model to the Lee–Carter model using the COM–poisson distribution.

Using the Generalized Autoregressive Score (GAS), put forth by Creal et al. (2008) and Creal et al. (2013), or the Dynamic Conditional Score model as proposed by Harvey (2013), we evaluate, project, and simulate mortality trends across various age groups. Within the GAS framework, the procedure for revising time-dependent parameters is driven by the score of the observable likelihood function.

This study proposes Generalized Autoregressive Score (GAS) model based on COM–Poisson distribution and compares its performance to Poisson, binomial, and negative binomial distributions as in the study conducted by Neves et al. (2017), which considered count variables. By implementing the suggested framework and employing a range of competing distributions, we are able to determine the most suitable outcome variable and the prime probability model for projecting mortality rates within the Lee–Carter model’s context.

One notable benefit of the framework we introduce, when juxtaposed with the traditional Lee–Carter model, is the ability to estimate parameters in a consolidated single step, as highlighted by (Neves et al. 2017). Moreover, projections of mortality rates are directly extracted from the designated model using Monte Carlo simulation, obviating the conventional reliance on other forecasting models, commonly ARIMA(0,1,1), as used in the standard Lee–Carter approach. Thus, the processes of parameter estimation, signal discernment, and forecasting are encompassed within one integrated model, as emphasized by (Creal et al. 2013). This distinctiveness sets our methodology apart from the Lee–Carter model and its variants, such as those discussed by Brouhns et al. (2002) and Renshaw and Haberman (2006). In essence, our method preserves the inferential validity that might be compromised in the multi-phased Lee–Carter model and several of its derivatives.

## The Lee–Carter mortality model

Lee and Carter (1992) proposed a single and efficient model for forecasting the central mortality rates:

1 $$\begin{aligned} \log (m_{xt}) = \alpha _x + \beta _x\kappa _t + \varepsilon _{xt} \end{aligned}$$

For a given set of ages $x$ ranging from 1 to $N$ and time points $t$ ranging from 1 to $T$:

- $m_{xt}$ denotes the central mortality rate for age $x$ at time $t$.
- $\kappa _t$ is a temporal parameter that embodies the shared trend in the logarithm of mortality rates across all ages.
- $\beta _x$ is a parameter specific to age $x$ and illustrates how sensitive the log of the mortality rate at that age is to the time trend depicted by $\kappa _t$.
- $\alpha _x$ acts as an intercept that is exclusive to age $x$.
- $\varepsilon _{xt}$ encompasses the elements not accounted for by the model, or the residuals. These are presumed to be independently and identically distributed with a normal distribution having a mean of 0 and a variance of $\sigma ^2$.

Also, one needs to impose the constraints: $\sum _{x=1}^{N}\beta _x = 1$ and $\sum _{t=1}^{T}\kappa _t = 0$, added to identify the model (Nigri et al. 2019). Using these constraints, one obtains the least squares estimator for $\alpha _x$, given by ${\hat{\alpha }}_x = \frac{1}{N}\sum _{x=1}^{N}\log (m_{xt})$.

The Lee–Carter mortality model can be distilled into three distinct stages, as detailed by (Giacometti et al. 2012).

1. In the initial estimation phase, the unknown parameters $\beta _x$ and $\kappa _t$ are deduced using Singular Value Decomposition (SVD) applied to the matrix formed by the centered age profiles ($\log (m_{xt}) - \alpha _x$) (Camarda and Basellini 2021).
2. The subsequent stage employs Ordinary Least Squares (OLS) to refine the fit of $\kappa _t$, the shared trend. This is achieved by minimizing discrepancies in the predicted number of deaths. Intrinsically, this adjustment gives heightened emphasis to ages witnessing higher death rates.
3. The final step is essential for forecasting the logarithm of mortality rates. This stage operates by preserving the previously estimated values for $\alpha _x$ and $\beta _x$ while projecting the time-dependent parameter $\kappa _t$. Conventionally, ARIMA models are employed for this forecasting purpose (Hong et al. 2021).

Subsequently, the mortality modeling framework developed by Lee–Carter was extended by Renshaw and Haberman (2008, 2006) by incorporating an additional age-cohort interaction term to capture the cohort effect:

2 $$\begin{aligned} \log (m_{xt}) = \alpha _x + \beta _x^{(1)} \kappa _t + \beta _x^{(0)} \gamma _{t-x} + \varepsilon _{xt} \end{aligned}$$

This study attempts to utilize both models (Lee–Carter and Renshaw-Haberman) to gain a more comprehensive understanding, building upon the research conducted previously by Rakhmawan et al. (2023).

## The Conway–Maxwell–Poisson distribution and its statistical properties

The Conway–Maxwell–Poisson (COM–Poisson) distribution extends the Poisson distribution with an extra parameter, $\nu$, allowing for flexible modeling of overdispersion or underdispersion. It can capture non-linear probability changes, connecting Negative binomial, Poisson, and Bernoulli distributions based on $\nu$. $\nu < 1$ indicates overdispersion, useful for modeling highly variable phenomena. Moments and sufficient statistics support various statistical analyses and modeling techniques.

### The Probability Function of the Conway–Maxwell–Poisson distribution

The COM–Poisson distribution is a versatile distribution that extends the Poisson distribution by accommodating overdispersion or underdispersion (Francis et al. 2012). Its probability function is defined as:

3 $$\begin{aligned} P(X=x) = \frac{{\lambda ^x \cdot e^{-\lambda }}}{{(x!)^\nu \cdot Z(\lambda , \nu )}}, \text { for } x = 0, 1, 2, \ldots \end{aligned}$$

In this equation, $\lambda$ represents the mean parameter of the distribution, and $\nu$ is an additional dispersion parameter that allows for flexible modeling. The term $Z(\lambda , \nu )$ ensures the normalization of the distribution (Gaunt et al. 2019). The COM–Poisson distribution satisfies the necessary conditions for a probability function, including non-negativity and the summation of probabilities equal to one.

One interesting aspect of the COM–Poisson distribution is the ability to capture non-linear changes in the ratio of successive probabilities. The ratio $P(X=x-1)/P(X=x)$ is given by $\frac{{x^{\nu -1}}}{{\lambda }}$. This feature allows for greater flexibility in modeling real-world phenomena where the probability of observing a certain event may not follow a linear pattern (Rasheed et al. 2022).

The series $\frac{{\lambda ^j}}{{(j!)^\nu }}$ in the probability function converges for any positive $\lambda$ and $\nu$ greater than zero, with one exception. As *j* approaches infinity, the ratio of two consecutive terms, $\frac{{\lambda }}{{j^\nu }}$, tends to zero, ensuring convergence.

The COM–Poisson distribution serves as a generalization of several well-known discrete distributions. When $\nu = 1$, the term $Z(\lambda , \nu )$ simplifies to $e^\lambda$, resulting in a standard Poisson($\lambda$) distribution. As $\nu$ tends towards infinity, $Z(\lambda , \nu )$ converges to $1+\lambda$, making the COM–Poisson distribution similar to a Bernoulli distribution with $P(X=1) = \frac{{\lambda }}{{1+\lambda }}$. On the other hand, when $\nu = 0$ and $\lambda < 1$, $Z(\lambda , \nu )$ represents a geometric sum, and the distribution becomes negative binomial. However, if $\nu = 0$ and $\lambda$ is not equal to 1, $Z(\lambda , \nu )$ does not converge, rendering the distribution undefined. This demonstrates how the COM–Poisson distribution acts as a continuous bridge, connecting the Negative binomial (with $\nu = 0$), Poisson (with $\nu = 1$), and Bernoulli (with $\nu = \infty$) distributions.

Values of $\nu$ less than 1 indicate flatter successive ratios compared to the Poisson distribution. Consequently, the COM–Poisson distribution exhibits longer tails and greater variability, a characteristic known as overdispersion. This property makes it a valuable tool for modeling phenomena where extreme events occur more frequently than predicted by a Poisson distribution (Mammadova and Özkale 2021).

### Moments of the distribution

The COM–Poisson distribution belongs to the family of two-parameter power series distributions (Borges et al. 2014). Moments of this distribution can be computed using the recursive formula:

4 $$\begin{aligned} E(X^{r+1}) = \lambda \cdot \frac{E\left( (X+1)^r\right) }{(1-\nu )}, \text { for } r = 0, 1, 2, \ldots \end{aligned}$$

This formula allows for the calculation of higher moments based on lower moments, making it convenient for analyzing the distribution’s properties. By recursively applying this formula, various moments such as the mean, variance, skewness, and kurtosis can be obtained.

To estimate the expected value *E*(*X*) of the COM–Poisson distribution, an asymptotic approximation for $Z(\lambda , \nu )$ can be utilized. This approximation, derived from Eq. (4) which is further explained in Appendix A, provides a close estimation:

5 $$\begin{aligned} E(X) \approx \frac{{\lambda ^{1/\nu - \nu ^{-1}}}}{{2\nu }} \end{aligned}$$

This approximation is particularly accurate when $\nu$ is close to 1 or when $\lambda$ is significantly larger than $10^\nu$ (Sellers et al. 2012).

### Sufficient statistics

In statistical inference, sufficient statistics play a crucial role in summarizing the information contained in a sample (Cox 2006). For the COM–Poisson distribution, the sufficient statistics are defined based on a set of *n* independent and identically distributed observations $x_1, x_2, \ldots , x_n$.

The likelihood function for $x_1, x_2, \ldots , x_n$ given the parameters $\lambda$ and $\nu$ can be expressed as:

6 $$\begin{aligned} L(x_1, x_2, \ldots , x_n | \lambda , \nu ) = \prod _{i=1}^{n} \left( \frac{{\lambda ^{x_i}}}{{(x_i!) \cdot Z^{-n}(\lambda , \nu )}}\right) \end{aligned}$$

This likelihood function combines the individual probabilities of observing each data point into a joint probability. By maximizing this likelihood function, parameter estimates can be obtained.

The factorization theorem states that the likelihood function can be factorized into functions of sufficient statistics. For the COM–Poisson distribution, the two sufficient statistics are $S_1 = \sum _{i=1}^{n} x_i$ (the sum of observations) and $S_2 = \sum _{i=1}^{n} \log (x_i!)$ (the sum of the logarithms of factorials of observations).

Moreover, Eq. (3) demonstrates that the COM–Poisson distribution belongs to the exponential family, making it amenable to various statistical analyses and modeling techniques commonly applied to exponential family distributions (Shmueli et al. 2005).

## The generalized auto-regressive score (GAS) model

The Generalized Autoregressive Score models, often referred to as dynamic conditional score models or score-driven models, were proposed by Harvey and Chakravarty (2008) and Creal et al. (2011). These models offer a comprehensive method for handling time series data.

Considering a time series denoted as $\{X_t\}_{t=1}^n$ with a sample size of *n*, one assumes a conditional density signified by the evolving parameter $\{f_t\}$, expressed as

7 $$\begin{aligned} P(x_t, \theta ) = P(x_t | f_{t-1}, \theta ), \quad t = 1, \ldots , n. \end{aligned}$$

In this context, $\theta$ represents a vector of hyperparameters. The term $f_{t-1}$ is associated with a parameter of the conditional distribution, often referred to as a filter, which evolves with time and is influenced by historical data. Within the framework of the GAS model, the progression of $f_t$ is determined by

8 $$\begin{aligned} f_{t+1} = \omega + \sum _{i=1}^{p} {A_{i} s_{t-i+1}} + \sum _{j=1}^{q} {B_{j} f_{t-j+1}} \end{aligned}$$

where the vector $\omega$ is a constant, while $A_i$ and $B_j$ are matrices that possess the suitable dimensions corresponding to $i = 1, \ldots , p$ and $j=1,\ldots , q$ respectively. The term $s_t$ stands for the scaled score, and typically, the inverse of the information matrix is employed.

GAS models have garnered significant interest, especially when utilized in the context of non-normal observations, as indicated by (Creal et al. 2013). For further applications of this methodology, one can refer to gasmodel.com.

## The GAS model for mortality case

Consider $y_{xt}$ as a generic outcome variable representing mortality in the context of mortality forecasting, where *x* denotes age or age group and *t* denotes time. Assuming that $y_{xt}$ has a conditional density or the probability mass function given by $p_x(y_{xt}|f_{t},{\mathcal {F}}_{t-1};\theta )$, drawing inspiration from the works of Lee and Carter (1992) and Creal et al. (2013), a factor model structure can be embraced. In this model, the $y_{xt}$ values at time instance *t* are deemed to be cross-sectionally independent. Therefore, given the time-varying parameter $f_{t}$ and the information set ${\mathcal {F}}_{t-1}$, the conditional distribution $p(y_{t}|f_{t},{\mathcal {F}}_{t-1};\theta )$ can be formulated as follows:

9 $$\begin{aligned} p(y_{t}|f_{t},{\mathcal {F}}_{t-1};\theta ) = \prod _{x=1}^{N} p_x(y_{xt}|f_{t},{\mathcal {F}}_{t-1};\theta ). \end{aligned}$$

In order to integrate the Lee–Carter model within the comprehensive parametric GAS framework, Neves et al. (2017) begin with the overarching representation for the progression of the logarithm of mortality rates as depicted in Eq. (1). If we make an assumption that the term $\kappa _{t}$ in this equation, which stands for the unified trend across all age categories, corresponds to the time-variant parameter of the suggested GAS model (i.e., $\kappa _{t} = f_{t}$), then a GAS(1,1) mechanism for $\kappa _{t}$ can be articulated in the subsequent manner:

10 $$\begin{aligned} \kappa _{t+1} = \omega + A s_{t} + B \kappa _{t}, \end{aligned}$$

where $s_{t}$ represents the scaled score of the likelihood, and $\omega$, *A*, and *B* are static unknown parameters. Notably, setting $B=1$ in Eq. (10) makes our updating mechanism similar to the original assumption made by Lee and Carter (1992).

Given an assumed probability model $p(y_{xt}|\kappa _{t},{\mathcal {F}}_{t-1};\theta )$ for the outcome variable $y_{xt}$, it is straightforward to derive the expressions for the scaled score $s_{t}$ and the information matrix $I_{t}$ as follows:

11 $$\begin{aligned} s_{t} = S_{t} \sum _{x=1}^{N} \nabla _{x_{t}}, \end{aligned}$$

where

12 $$\begin{aligned} \nabla _{x_{t}}= & \frac{\partial }{\partial \kappa _{t}} \log p_x(y_{xt}| \kappa _{t}, {\mathcal {F}}_{t-1}; \theta ), \end{aligned}$$

13 $$\begin{aligned} S_{t}= & I_{t}^{-1/2}, \end{aligned}$$

with

14 $$\begin{aligned} I_{t} = \sum _{x=1}^{N} I_{xt}, \end{aligned}$$

and

15 $$\begin{aligned} I_{xt} = E_{t-1} \left[ \nabla _{xt} \nabla _{xt}^{\prime } \right] . \end{aligned}$$

For every age denoted by *x*, deriving the logarithm from the probability model as shown in Eq. (12) gives us the ’partial’ score. From this, by utilizing Eq. (15), we can deduce the expected value of $\nabla _{xt} \nabla _{xt}^{\prime }$, leading to the formation of the ’partial’ information matrix. Given these formulations, the scaled score matrix, represented as $S_{t}$ in Eq. (13), can be determined directly.

In conclusion, when selecting a specific form for $p_x(y_{xt}|\kappa _{t},{\mathcal {F}}_{t-1};\theta )$ and taking into account the assumptions presented in Eq. (9), the likelihood function can be articulated in the ensuing manner:

16 $$\begin{aligned} L(\theta ) = \prod _{t=1}^{T} \prod _{x=1}^{N} p_x(y_{xt}|f_{t},{\mathcal {F}}_{t-1};\theta ). \end{aligned}$$

Hence, the logarithm of the likelihood is given by:

17 $$\begin{aligned} l(\theta ) = \sum _{t=1}^{T} \sum _{x=1}^{N} \log p_x(y_{xt}|f_{t},{\mathcal {F}}_{t-1};\theta ). \end{aligned}$$

To estimate the vector of static unknown parameters $\theta$, we maximize the log-likelihood function using appropriate nonlinear optimization algorithms such as Broyden-Fletcher-Goldfarb-Shanno or Berndt-Hall-Hall-Hausman (Ratnasari et al. 2022; Zhao 2021; León et al. 2005; Head and Zerner 1985).

It should be emphasized that each specific selection of $p_x(y_{xt}|\kappa _{t},{\mathcal {F}}_{t-1};\theta )$ leads to a distinct updating formula for the shared trend as shown in Eq. (10) and a unique likelihood function denoted by $l(\theta )$. This differentiation becomes evident in Sections 5.1$-$5.3 where particular structures for $p_x(y_{xt}|\kappa _{t},{\mathcal {F}}_{t-1};\theta )$ are considered. Additionally, it’s crucial to point out that the update procedure for $\kappa _{t}$ is designed in a manner that factors in the age-dependent parameters $\beta _{x}$ to emphasize the sole time-dependent parameter, along with components critical for achieving the scaled score at instance *t*.

For the purpose of estimating the static parameter vector $\theta$, the log-likelihood function depicted in Eq. (17) is maximized. Hence, within the GAS adaptation of the Lee–Carter model, the process of estimating parameters is streamlined to a singular phase. Projections spanning multiple steps for both time-varying parameters and upcoming data points can be derived via Monte Carlo techniques, referencing the recursion shown in Eq. (10) (Gao and Shi 2023; Creal et al. 2013). This methodology maintains pertinent inferential outcomes which might be omitted in the foundational Lee–Carter model and its subsequent versions. It ensures the identified components remain pertinent to the desired outcome variables throughout the estimation and projection phases (Neves et al. 2017).

To model mortality rates using variations of the Lee–Carter model, we assume, as in its original formulation, that the force of mortality rates for any age group *x* at time *t* follows the relation:

18 $$\begin{aligned} \mu _{(x+c)t} = \mu _{xt} \quad \text {for} \quad 0 \le c < g, \end{aligned}$$

where *g* represents the width of the age group. Consequently, the force of mortality rates is equal to the central mortality rates ($\mu _{xt} = m_{xt}$).

Given that the GAS likelihood function is presented in a closed form, it allows us the flexibility to choose varied probability distributions for the target variable $y_{xt}$. This facilitates the expansion of the Lee–Carter model beyond just the lognormal distribution. In subsequent sections, we introduce and elaborate on the COM–Poisson GAS models tailored for mortality predictions, juxtaposing their outcomes with those from Poisson, Binomial, and Negative Binomial distributions, akin to the study conducted by Neves et al. (2017).

### Poisson GAS model for the number of deaths

This primary model presented concentrates on death counts ($d_{xt}$) at a certain age *x* and time *t*, viewed as separate events of a Poisson random variable. This concept operates under the premise that the population at risk ($L_{xt}$) is known, which is a typical scenario in real-world data scenarios. Hence, the statistical equation can be described as:

19 $$\begin{aligned} p(d_{xt} | L_{xt}, {\mathcal {F}}_{t-1}) \sim \text {Poisson}(\lambda _{xt}), \end{aligned}$$

with the rate parameter expressed as:

20 $$\begin{aligned} \lambda _{xt} = L_{xt} \exp (\alpha _x + \beta _x \kappa _t). \end{aligned}$$

Considering the mortality rate as $m_{xt} = \frac{d_{xt}}{L_{xt}}$, and acknowledging the population at risk ($L_{xt}$), the average and fluctuation of the mortality rate can be stated as:

$$\begin{aligned} & E(m_{xt} | L_{xt}, {\mathcal {F}}_{t-1}) = \exp (\alpha _x + \beta _x \kappa _t), \\ & \quad \text {Var}(m_{xt} | L_{xt}, {\mathcal {F}}_{t-1}) = \frac{1}{L_{xt}} \exp (\alpha _x + \beta _x \kappa _t). \end{aligned}$$

For the GAS (1, 1) update process (referenced as Eq. (10)), the "partial" score and details, using the Poisson framework, can be articulated as $\nabla _{xt} = \frac{\partial \log p_{x}(d_{xt} | L_{xt}, F_{t-1})}{\partial \kappa _t} = \beta _x (d_{xt} - \lambda _{xt})$ and $I_{xt} = E_{t-1}[\nabla _{xt} \nabla '{xt}] = \beta ^2_x \lambda {xt}$, respectively.

### Binomial GAS model for the number of deaths

In the second GAS model, attention remains on the death count ($d_{xt}$) as the principal metric. Distinctively in this variant, $d_{xt}$ is modeled to adhere to a binomial distribution, contingent on the population count at the start of the year ($l_{xt}$):

21 $$\begin{aligned} p(d_{xt} | l_{xt}, F_{t-1}) \sim \text {Bin}(l_{xt}, q_{xt}), \quad 0 < q_{xt} \le 1, \end{aligned}$$

Here, $q_{xt}$ denotes the age-specific mortality probability at time *t*, interconnected with the general trend $\kappa _t$ via a logistic function:

$$\begin{aligned} q_{xt} = \frac{1}{1 + e^{-\exp (\alpha _x + \beta _x \kappa _t)}}. \end{aligned}$$

When considering the initial population size ($l_{xt}$) and under the binomial framework, the mortality rate’s mean and variability are formulated as:

$$\begin{aligned} & E(m_{xt} | l_{xt}, {\mathcal {F}}_{t-1}) = \frac{l_{xt}}{L_{xt}} \frac{\exp (\alpha _x + \beta _x \kappa _t)}{\left( 1 + \exp (\alpha _x + \beta _x \kappa _t)\right) }, \\ & \quad \text {Var}(m_{xt} | l_{xt}, {\mathcal {F}}_{t-1}) = \frac{l_{xt}}{L_{xt}^2} \frac{\exp (\alpha _x + \beta _x \kappa _t)}{\left( 1 + \exp (\alpha _x + \beta _x \kappa _t)\right) ^{2}}. \end{aligned}$$

In parallel to the Poisson model, for the execution of the GAS (1, 1) mechanism to update $\kappa _t$, it’s essential to calculate both the "partial" score and the "partial" information, delineated as $\nabla _{xt} = \beta _x (d_{xt} - l_{xt} q_{xt})$ and $I_{xt} = \beta ^2_x l_{xt} q_{xt} (1 - q_{xt})$, respectively.

### Negative binomial GAS model for the number of deaths

Delwarde et al. (2007) have recommended a novel approach to address the over-dispersion often observed in mortality data. Their suggestion involves utilizing a negative binomial distribution to model the number of deaths, taking into account the population exposed to risk ($L_{xt}$). In light of this, we propose a modified approach: a negative binomial GAS model:

22 $$\begin{aligned} p(d_{xt} | L_{xt}, {\mathcal {F}}_{t-1}) \sim \text {NB}(r_x, h_{xt}). \end{aligned}$$

This model introduces an additional parameter ($r_x$), specific to each age group and constant over time, enhancing the model’s adaptability and performance in data fitting, a significant improvement over the Poisson GAS model.v

Aligning with the approaches in the Poisson and binomial models, the expectation and variance of the mortality rate, conditional on the exposed population ($L_{xt}$), are inferred as follows:

$$\begin{aligned} E(m_{xt} | L_{xt}, {\mathcal {F}}_{t-1}) &= \exp (\alpha _x + \beta _x \kappa _t) = \frac{r_x (1 - h_{xt})}{L_{xt} h_{xt}}, \\ \text {Var}(m_{xt} | L_{xt}, {\mathcal {F}}_{t-1}) &= \frac{r_x (1 - h_{xt})}{(L_{xt} h_{xt})^2} = \frac{\exp (\alpha _x + \beta _x \kappa _t)}{L_{xt} h_{xt}}. \end{aligned}$$

For the GAS (1, 1) updating mechanism, essential calculations include the "partial" score and "partial" information. These are expressed as $\nabla _{xt} = \beta _x [(d_{xt} h_{xt}) - (1 - h_{xt}) r_x]$ and $I_{xt} = \beta ^2_x (1 - h_{xt}) r_x$. These are integral for the effective implementation of the GAS (1, 1) model.

### COM–Poisson GAS model for the number of deaths

The COM–Poisson probability distribution function, a versatile statistical model, can be characterized by the equation:

23 $$\begin{aligned} P(Y=y) = \frac{\lambda ^y}{(y!)^vZ(\lambda ,v)}, \end{aligned}$$

where

$$\begin{aligned}y=0,1,2,\ldots ,\lambda > 0, v \ge 0\end{aligned}$$.

This defines the probability for a variable *Y*, with $Z(\lambda ,v) = \sum _{s=0}^{\infty } \frac{\lambda ^s}{(s!)^v}$ acting as a normalization factor. The parameter *v*, known as the dispersion parameter, alters the distribution’s characteristics: values of $v > 1$ indicate under-dispersion, while $v < 1$ suggest over-dispersion. The COM–Poisson distribution extends beyond the Poisson distribution (for $v=1$) to include the negative binomial (for $v=0;\lambda <1$) and binomial distributions (as $v \rightarrow \infty$ with a probability of $\lambda / (1+\lambda )$), for more details see Jamal et al. (2021); Mahmood et al. (2019).

Although direct correlations between $\lambda$, *v*, and moments are not straightforward, certain relationships elucidate the influence of these parameters on the distribution. An example is the expression $\lambda =E(Y^v)$, which associates $\lambda$ with the expected value of the variable *Y* raised to the power of *v*, as detailed in (Sellers et al. 2012).

In situations where $v\le 1$ or $\lambda >10^v$, approximations for the expected value and variance are:

24 $$\begin{aligned} & E(Y) \approx \lambda ^{1/v} - \frac{v-1}{2v} \end{aligned}$$

25 $$\begin{aligned} & \quad \text {Var}(Y) \approx \frac{1}{v} \lambda ^{1/v} \end{aligned}$$

In the context of modeling mortality rates, where the number of deaths is a key variable and $\lambda _{xt} = L_{xt} \cdot e^{\alpha _x + \beta _x\kappa _t}$, the COM–Poisson GAS model provides insights into mortality trends. For $v\le 1$ or $\lambda >10^v$, the mean and variance of the mortality rate ($m_{xt}=\frac{d_{xt}}{L_{xt}}$) are:

$$\begin{aligned} & E(m_{xt}|L_{xt} {\mathcal {F}}_{t-1}) = {(L_{xt} \cdot e^{\alpha _x + \beta _x\kappa _t})}^{1/v} - \frac{v-1}{2v} \\ & \quad \text {Var}(m_{xt}|L_{xt} {\mathcal {F}}_{t-1}) = \frac{1}{v} \cdot {(L_{xt} \cdot e^{\alpha _x + \beta _x\kappa _t})}^{1/v} \end{aligned}$$

When focusing on mortality rates, the partial score and information matrix for the GAS(1,1) model, derived in Appendix A, are identified as $\nabla _{xt} = \beta _x \cdot (d_{xt} - \lambda _{xt})$ and $I_{xt} = \beta _x^2 \cdot \lambda _{xt}$, respectively.

### Summary of GAS models for the mortality

**Table 1:** Summary of the distributions and their properties

Probability model ($Y = d_{xt}$)	Partial score ($\nabla _{xt}$)	Partial information matrix ($I_{xt}$)
COM–Poisson		
$P(\lambda _{xt}, \nu ) = \frac{{\lambda _{xt}^{d_{xt}}}}{{d_{xt}^{\nu } \cdot Z(\lambda _{xt}, \nu )}}$	$\beta _x \cdot \left( d_{xt} - \lambda _{xt} + \frac{{\nu -1}}{{2\nu }} \right)$	$\beta _x^2 \cdot \lambda _{xt}$
Poisson		
$P(\lambda _{xt}) = \frac{e^{-\lambda _{xt}} \cdot \lambda _{xt}^{d_{xt}}}{d_{xt}!}$	$\beta _x \cdot \left( d_{xt} - \lambda _{xt} \right)$	$\beta _x^2 \cdot \lambda _{xt}$
Binomial		
$P(l_{xt}, q_{xt}) = q_{xt}^{y_{xt}} \cdot (1-q_{xt})^{l_{xt}-y_{xt}}$	$\beta _x \cdot \left( d_{xt} - l_{xt} \cdot q_{xt} \right)$	$\beta _x^2 \cdot l_{xt} \cdot q_{xt} \cdot (1-q_{xt})$
Negative binomial		
$$P(r_{x}, h_{xt}) = \left( {\begin{array}{c}y_{xt}+r_{x}-1\\ y_{xt}\end{array}}\right) \cdot (1-h_{xt})^{y_{xt}} \cdot {h_{xt}}^{r_{x}}$$	$\beta _x \cdot [d_{xt} \cdot h_{xt} - (1-h_{xt}) \cdot r_{x}]$	$\beta _x^2 \cdot (1-h_{xt}) \cdot r_{x}$

Our developed Generalized Autoregressive Score (GAS) models exhibit a unique statistical trait that significantly enhances their adaptability in data fitting: the dynamic evolution of both the conditional mean and variance of mortality rates across different age groups. A notable aspect of the COM–Poisson model within this framework is its capacity for automatic updates of the $\nu$ parameter. This feature allows the model to embody a Poisson distribution when $\nu$ equals 1 and transition to other distributions like the negative binomial or binomial for values of $\nu$ at 0 or infinity. This flexibility marks a departure from the fixed constraints noted in the work of Lee and Carter (1992) and further expanded upon in (Blasques et al. 2014).

An illustrative Table 1 is included to summarize these diverse GAS models. These models, particularly the discrete distribution ones like Poisson, binomial, and negative binomial, were initially conceptualized for assessing the number of deaths ($d_{xt}$) for specific age *x* and time *t*, as expounded in subsections 5.1 to 5.3, and also in the context of the COM–Poisson distribution in subsection 5.4. Nevertheless, these models are adaptable for calculating the mortality rates ($m_{xt}$), taking into account the exposed population ($L_{xt}$) in the cases of the Poisson and negative binomial models, and the population size at the year’s onset ($l_{xt}$) for the binomial model. Utilizing any of these proposed GAS models, which cater to both discrete and continuous distributions, allows for the effective prediction of death rates across various age demographics.

### Model evaluation

This study attempts to explore a holistic approach to mortality model evaluation by considering various aspects such as in-sample fit, model complexity, and out-of-sample prediction accuracy. The ultimate goal is to address common challenges in mortality modeling, including over-parameterization and overfitting, while considering the balance between good generalizability and model interpretation.

First, the generalized model has good and simple properties, so it can provide an adequate description of the main features of the data and thus avoid over-parameterization by ensuring that the final model provides an adequate description without too many parameters. By following Mahmood (2020); Mahmood et al. (2021), the Bayes Information Criterion (BIC) and Akaike Information Criterion (AIC) are then used as tools to select the optimal model, taking into account the balance between in-sample fit and model complexity, using following formula:

26 $$\begin{aligned} BIC = -2 \text {LL} + n_p \ln (n_d) \end{aligned}$$

27 $$\begin{aligned} AIC = -2 \text {LL} + 2n_p \end{aligned}$$

where *LL* is the estimated log likelihood, $n_p$ is the effective number of parameters (the number of parameters minus the number of identification constraints) and $n_d$ is the number of observations. Lower values of these measures is preferred.

Secondly, an out-of-sample prediction accuracy evaluation is conducted using the mean absolute percentage error (MAPE) with formula:

28 $$\begin{aligned} MAPE_i = \frac{1}{N_i} \sum _{x,t} \frac{| {\hat{m}}_{xt,i} - m_{xt,i} |}{m_{xt,i}} \end{aligned}$$

where $N_i$ is the number of observations in each population which is the number of ages or age classes times the number of years. MAPE is a useful metric for assessing the extent to which the mortality model in this study fits data that has not been seen before. In addition, this study also analyzed descriptive statistics such as the mean, median, minimum, and maximum values of the MAPE of various results to provide a holistic picture of the model’s performance.

## Applications

**Fig. 1:**
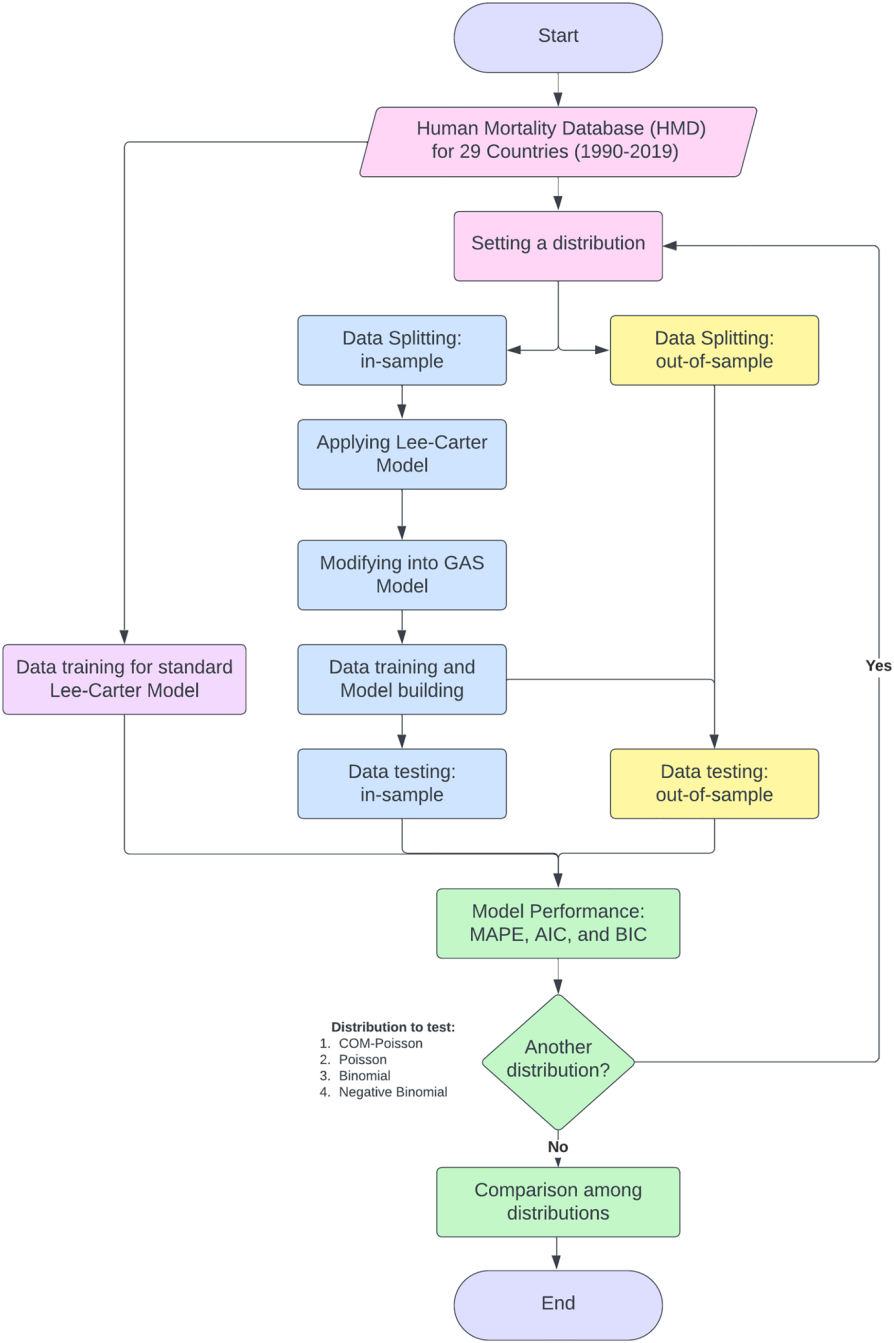
A flow diagram about the implementation of proposed model

The data utilized in this study was sourced from the Human Mortality Database (HMD) (Jdanov et al. 2019) encompassing 29 countries, spanning the years 1990 to 2019. The data was divided into two segments, namely the in-sample and out-of-sample with three scenarios of splitting, facilitating the application of the Generalized Autoregressive Score (GAS) model across the 29 countries. For each country, the dataset included all age groups from 0 years to 90+ years, as some countries only provided mortality data in grouped age format. The study aimed to provide guidance for these countries, although the HMD already provides single-age data. Additionally, grouping the data reduced the potential for non-linear parameter trends, resulting in improved parameter estimation (Schielzeth et al. 2020). The initial analysis focused on data from some countries to build upon the research conducted by Neves et al. (2017). In general, the workflow conducted in this study, as illustrated in Fig. 1, indicates that there are typically four iterations involved in constructing GAS models to build models based on the COM–Poisson, Poisson, Binomial, and Negative Binomial distributions. Furthermore, at the conclusion, in addition to the models derived from these four distributions, this research also compares the outcomes with the standard Lee–Carter model.

**Table 2:** AIC and BIC for the different GAS models

Model	Split 1		Split 2		Split 3	
	AIC	BIC	AIC	BIC	AIC	BIC
COM–Poisson	11,748	12,483	11,358	12,158	12,475	13,273
Poisson	29,374	29,423	28,763	28,896	29,947	30,125
Binomial	31,694	32,948	31,368	32,483	32,449	33,189
Negative Binomial	12,956	12,994	12,368	12,527	13,295	13,429
Lee–Carter	27,340	28,574	26,182	27,565	31,628	32,941
Renshaw–Haberman	27,121	29,023	25,538	27,698	31,340	33,376

Three splitting scenarios for model building are shown in Table 2. The first splitting uses 1990–2004 data as in sample; the second splitting uses 1990–2009 as in sample; and the third splitting uses 1990–2014 as in sample. The results of the three splits show that split scenario 2 is the most optimal split because it shows the minimum AIC and BIC values. Moreover, the result in Table 2 demonstrates that when evaluating the AIC and BIC values of each model fitted to the number of deaths, it can be concluded that among the models with discrete distributions (Poisson, Binomial, and Negative Binomial), the COM–Poisson distribution emerges as the optimal choice, minimizing both the AIC and BIC. This finding can be attributed to the inclusion of an additional static parameter $\nu$, which enhances the model fitting performance without significantly increasing its complexity (Luengo et al. 2020). The research from Neves et al. (2017) compare these models (Poisson, Binomial, and Negative binomial) to Gaussian and Beta. However, a direct comparison of these four AIC and BIC values with the other two distributions (Gaussian and Beta) is not appropriate due to the different nature of continuous distributions (Crotty and Holland 2022). The Gaussian model is fitted to log mortality rates, while the Beta model is fitted to mortality rates.

**Table 3:** MAPE in-sample (1990–2009) for 29 countries

Model	Min	$Q_1$	Mean	$Q_3$	Max
COM–Poisson	0.008	1.376	3.297	4.385	10.253
Poisson	0.011	2.173	3.407	4.752	10.384
Binomial	0.022	1.493	3.872	5.582	11.048
Neg. binomial	0.042	1.918	4.937	5.048	10.593
Lee–Carter	0.013	1.128	3.384	4.374	10.034
Renshaw–Haberman	0.001	1.037	3.285	4.194	10.001

In addition to using AIC and BIC measures, the superiority of the COM–Poisson model is also evident from the mean absolute percentage error (MAPE) metrics, both in the in-sample and out-of-sample analyses. Table 3 and 4 present the MAPE values for the four probabilistic models (COM–Poisson, Poisson, Binomial, Negative Binomial) during two different periods: In Sample (1990–2009) and Out Sample (2010–2019). Table 3 is the simulation result of the MAPE in-sample (1990–2009) for 29 countries.

In the in-sample analysis, it can be observed that the Poisson model (COM–Poisson with $\nu =1$) exhibits a low mean MAPE of 3.4 percent, followed by the Binomial model at 3.9 percent. The Negative Binomial model has mean MAPEs of 4.9 percent. Among all models, the COM–Poisson model demonstrates the lowest mean, Q1, Q3, min, and max values, indicating a tighter and more consistent distribution of errors. These results suggest that the COM–Poisson and Poisson models are the most accurate for the in-sample analysis.

**Table 4:** MAPE out-of-sample (2009–2019) for 29 countries

Model	Min	$Q_1$	Mean	$Q_3$	Max
COM–Poisson	0.017	2.337	6.176	8.138	21.435
Poisson	0.026	2.725	6.431	8.950	35.553
Binomial	0.106	4.808	8.826	11.174	35.541
Neg. binomial	0.065	3.612	7.085	9.596	23.148
Lee–Carter	0.216	2.364	6.395	8.734	24.485
Renshaw–Haberman	0.104	2.937	5.684	7.375	20.394

Moving to the out-of-sample analysis, this study attempted to forecast deaths using the training data for all models and compare the forecasted data with the out-of-sample data. The result of COM–Poisson model continues to exhibit the lowest mean MAPE of 6.1 percent, followed by the Poisson, Negative Binomial, and Binomial models at 6.4 percent, 7.1 percent, and 8.8 percent, respectively.

The COM–Poisson model maintains the lowest values, indicating a relatively consistent distribution of errors (Dennis et al. 2019). The Poisson and Negative Binomial models have slightly similar values, suggesting a slightly wider range of errors. On the other hand, the Binomial model demonstrates a much higher value, indicating a higher proportion of extremely large errors. These results indicate that the COM–Poisson model remains the most accurate, while the Binomial models are the least accurate.

To confirm the superiority of the COM–Poisson model, a Diebold-Mariano (DM) test was conducted using the MAPE loss function (Zhou et al. 2021). The test results do not reject the notion that the COM–Poisson model is more accurate than the other competing models. Furthermore, the previous explanations reveal that the COM–Poisson distribution, particularly when the value of $\nu$ is extremely close to or equal to 1, closely approximates the Poisson distribution with similar metric outcomes.

Based on these findings, it can be argued that COM–Poisson serves as a generalized distribution encompassing other distributions by modifying the value of $\nu$. The modification of $\nu$ depends on the central tendency of the data, whether it exhibits equidispersion, underdispersion, or overdispersion.

Both tables of in-sample and out-of-sample MAPE highlight the inclusion of the COM–Poisson distribution in mortality data simulations, as it outperforms other distributions, especially when compared to the Poisson, Binomial, and Negative Binomial distributions, which are primarily used to model count data outcomes. Remarkably, the COM–Poisson distribution encompasses both the Poisson and Negative Binomial distributions. Therefore, the COM–Poisson distribution proves suitable for modeling death in distributions with count data outcomes.

**Table 5:** In-sampe (IS) and Out-of-sample (OS) MAPE of Australian GAS models

	COM–Poisson		Poisson		Binomial		Neg. Binomial		Lee–Carter		Renshaw-Haberman	
Age Group	IS	OS	IS	OS	IS	OS	IS	OS	IS	OS	IS	OS
0	6.8	19.3	6.3	21.0	7.6	21.6	5.0	20.1	6.9	21.9	5.4	20.7
1–4	5.4	17.4	6.2	18.1	6.6	18.8	5.8	18.0	6.4	19.3	6.2	18.7
5–9	4.3	14.0	4.3	14.3	4.1	14.4	4.7	13.7	4.7	15.4	5.1	15.3
10–14	3.3	11.7	3.9	12.3	4.0	12.6	3.1	11.9	4.0	13.4	3.4	13.0
15–19	2.9	9.3	2.1	9.7	3.2	9.4	2.5	9.7	2.9	10.2	2.7	10.0
20–24	0.4	1.1	1.0	3.0	1.0	2.9	0.4	1.2	0.8	2.2	0.5	1.1
25–29	0.9	2.6	1.7	5.0	1.8	5.3	0.9	2.9	1.4	4.1	0.9	2.7
30–34	3.0	10.0	2.6	11.2	3.7	10.9	3.3	10.3	3.2	10.9	3.4	10.2
35–39	3.9	12.7	3.4	13.0	4.4	14.5	4.1	13.5	4.0	13.7	4.1	12.9
40–44	6.0	17.6	3.2	18.1	5.3	18.5	5.0	16.7	4.9	17.9	5.0	17.8
45–49	4.4	12.5	2.8	13.3	4.4	13.0	4.3	13.4	4.1	13.5	4.4	12.9
50–54	3.0	11.4	4.3	12.2	4.3	12.7	4.2	12.0	4.1	12.7	4.4	12.0
55–59	3.8	11.8	2.7	10.1	2.9	12.3	3.6	10.1	3.4	11.7	3.8	12.5
60–64	0.4	1.6	0.5	2.4	0.8	2.7	0.5	1.8	0.6	2.2	0.6	1.6
65–69	0.9	3.4	0.9	4.2	1.1	3.6	1.2	4.3	1.0	3.9	1.2	3.4
70–74	2.8	10.1	2.5	10.3	3.7	11.9	3.3	10.8	2.9	10.3	3.2	9.6
75–79	2.4	9.0	3.2	10.4	3.2	10.3	2.8	9.6	2.5	8.4	2.4	7.7
80–84	2.3	6.9	2.8	9.7	2.6	7.8	2.1	7.4	1.8	5.7	1.5	5.0
85–89	5.2	14.8	4.6	15.2	5.2	16.7	4.5	15.7	2.9	9.2	2.7	8.8
90+	2.5	8.2	3.1	9.1	3.0	9.5	3.1	9.2	1.2	3.6	1.3	3.3
Total	2.9	10.3	4.4	11.0	3.8	11.2	3.5	10.8	3.2	10.5	3.1	10.0

**Fig. 2:**
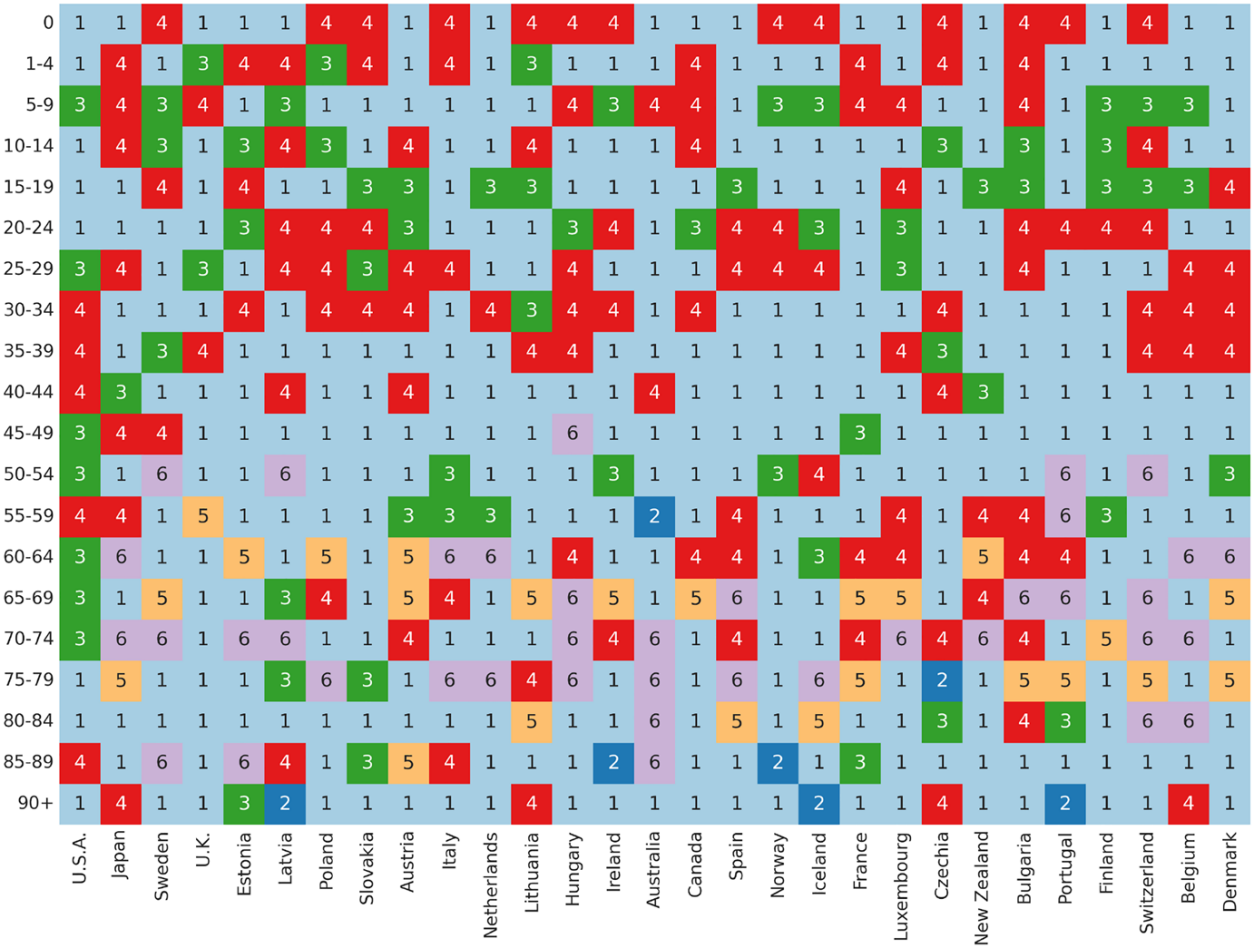
Heatmap of the most minimum MAPE among models, with coded (1) COM–Poisson, (2) Poisson, (3) Binomial, (4) Negative Binomial, (5) Lee–Carter, and (6) Renshaw-Haberman

In order to achieve a comprehensive understanding of error metrics, a thorough analysis was conducted to scrutinize the MAPE generated by the models, categorizing them into distinct age groups and countries, which constituted the primary focus of this study. The MAPE outcomes for each age group and country are presented in Fig. 2. This figure effectively portrays the distribution of the lowest MAPE values among all models, underscoring the dominance of the COM–Poisson model as depicted by its color-coded representation in the tabular mapping. These results strongly suggest that the COM–Poisson model yields the lowest MAPE values when compared to alternative distributions.

**Fig. 3:**
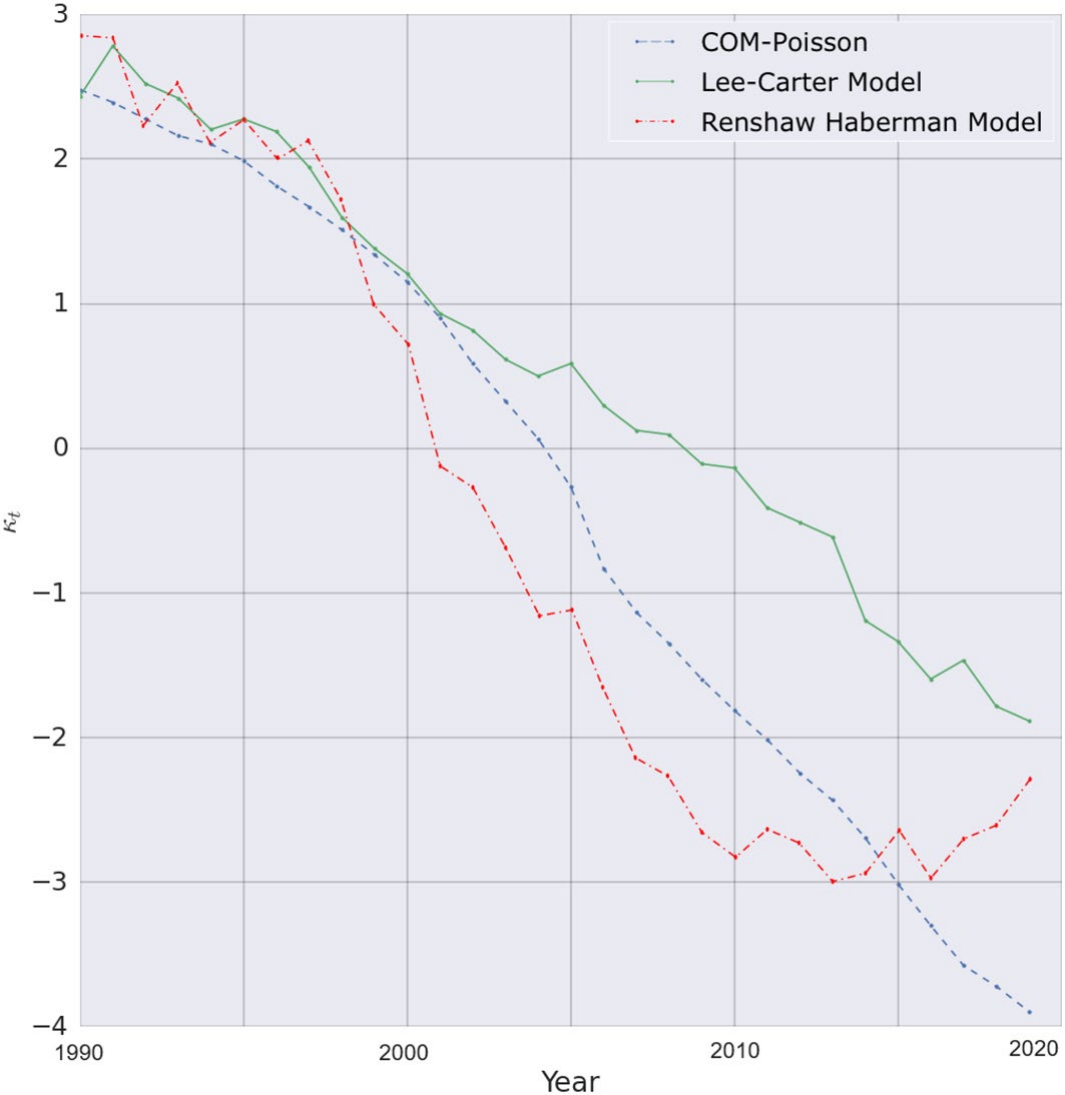
Time series of the mortality common trend for the Australian data ($\kappa _t$) estimated by the COM–Poisson GAS, Lee–Carter, and Renshaw–Haberman model

As a practical example, the MAPE data for Australia during both in-sample and out-of-sample periods are concisely presented in Table 5. The consistent findings from these comparisons distinctly demonstrate the superiority of the COM–Poisson model over other models, as evinced by the MAPE values obtained for both In-sample and Out-of-sample periods. Moreover, to holistically assess the performance of the Generalized Autoregressive Score (GAS) Model with the Lee–Carter model, a comparative analysis of their respective parameters was undertaken. This comparative investigation primarily focused on the year-related or common parameters, namely Kappa. Figure 3 graphically illustrates the comparison of how the common parameters in the GAS model, Lee–Carter, and the Renshaw-Haberman model estimated their outcomes over the period from 1990 to 2019. Notably, the COM–Poisson model within the GAS framework exhibits a smoother trend when contrasted with the other models, attributed to the latter’s recurrent re-estimation of the common trend to minimize errors arising from the number of deaths within each age group (Rabbi and Mazzuco 2021).

In the realm of mortality time series forecasting, the ability of a model to effectively capture volatility is of paramount importance. In this context, the COM–Poisson GAS model emerges as a superior performer, adept at capturing the volatility of mortality when compared to the Lee–Carter model. The COM–Poisson model comprehensively incorporates the overdispersion of mortality data into its model formulation, thereby enhancing its forecasting capabilities (Mustafa et al. 2023).

Notwithstanding the Lee–Carter model’s status as a widely utilized standard in mortality analysis, its three-step approach to estimating and projecting mortality rates necessitates careful consideration of its potential impact on resultant errors. To corroborate this assertion, this study thoroughly investigated the application of the Lee–Carter model across diverse age groups within Australian mortality data. The calculated Mean Absolute Percentage Error (MAPE) for each age group yielded an average of 3.48 percent for the in-sample period (comprising existing data) and 13.19 percent for the out-of-sample period (encompassing data yet to be observed during modeling).

## Conclusion

This research endeavors to develop the Generalized Autoregressive Score (GAS) model as a predictive tool for mortality rates across various countries, utilizing a versatile dispersion application through the COM–Poisson distribution. This model serves as an extension of the renowned and widely used Lee–Carter mortality model. Leveraging the COM–Poisson distribution aims to enhance the model’s flexibility and robustness in parameter estimation.

A noteworthy feature of the GAS model is its ability to generate one-step forecasts, distinguishing it from the three-step requirement of the Lee–Carter model. Through rigorous comparisons with various count variable distributions such as Poisson, binomial, and negative binomial, the COM–Poisson distribution exhibited outstanding performance in predicting mortality rates.

The implementation of the GAS model involved mortality data from 29 countries spanning the years 1990 to 2019. Following extensive testing, the COM–Poisson model demonstrated superior suitability for mortality rate forecasting in these countries.

The flexibility inherent in the GAS framework offers opportunities for further model development and refinement. In conclusion, this study substantiates that the COM–Poisson GAS model holds immense potential as a robust tool for analyzing mortality time series, especially when confronted with time-varying parameters and non-conventional data distributions.

## Data Availability

The datasets generated during and/or analyzed during the current study are available from the corresponding author upon reasonable request.

## Appendix A COM–Poisson mortality model

### A.1 Partial score ($\nabla _{xt}$)

To derive $\frac{d}{d\kappa _t} \log P(X=d_{xt})$ with respect to $\kappa _t$, we need to differentiate the expression $\log P(X=d_{xt})$ with respect to $\kappa _t$.

First, let’s express $\lambda _{xt}$ in terms of $L_{xt}$, $\alpha _x$, $\beta _x$, and $\kappa _t$:

$$\begin{aligned} \lambda _{xt} = L_{xt} \cdot e^{\alpha _x + \beta _x \cdot \kappa _t} \end{aligned}$$

Now, let’s write the expression for $\log P(X=d_{xt})$:

$$\begin{aligned} \log P(X=d_{xt}) = \log \left[ \frac{{\lambda _{xt}^{d_{xt}}}}{{d_{xt}^{\nu } \cdot Z(\lambda _{xt}, \nu )}}\right] \end{aligned}$$

Using logarithmic properties, we can simplify this expression:

$$\begin{aligned} \log P(X=d_{xt}) = d_{xt} \cdot \log \lambda _{xt} - \nu \cdot \log (d_{xt}) - \log Z(\lambda _{xt}, \nu ) \end{aligned}$$

Now, we can differentiate $\log P(X=d_{xt})$ with respect to $\kappa _t$:

$$\begin{aligned} & \frac{d}{d\kappa _t} \log P(X=d_{xt}) = \\ & \quad \frac{d}{{d\kappa _t}} \left( d_{xt} \cdot \log \lambda _{xt} - \nu \cdot \log (d_{xt}) - \log Z(\lambda _{xt}, \nu ) \right) \end{aligned}$$

To simplify the differentiation, we need to consider that $\lambda _{xt}$ depends on $\kappa _t$:

$$\begin{aligned} & \frac{d}{d\kappa _t} \log P(X=d_{xt}) = \\ & \quad \frac{d}{{d\kappa _t}} \left( d_{xt} \log \left( L_{xt} \cdot e^{\alpha _x + \beta _x \cdot \kappa _t} \right) - \right) \\ & \quad \nu \cdot \log (d_{xt}) - \log Z\left( \left( L_{xt} \cdot e^{\alpha _x + \beta _x \cdot \kappa _t} \right) , \nu \right) \end{aligned}$$

Now, firstly, let’s differentiate each term separately:

A1 $$\begin{aligned} & \frac{d}{{d\kappa _t}} \left( d_{xt} \log \left( L_{xt} \cdot e^{\alpha _x + \beta _x \cdot \kappa _t} \right) \right) = d_{xt} \cdot \frac{1}{{L_{xt} \cdot e^{\alpha _x + \beta _x \cdot \kappa _t}}} \cdot L_{xt} \cdot \beta _x \cdot e^{\alpha _x + \beta _x \cdot \kappa _t} \nonumber \\ & \quad \frac{d}{{d\kappa _t}} \left( d_{xt} \log \left( L_{xt} \cdot e^{\alpha _x + \beta _x \cdot \kappa _t} \right) \right) = d_{xt} \cdot \beta _x \end{aligned}$$

Secondly, since $\log (d_{xt})^\nu$ does not depend on $\kappa _t$, its derivative is zero with respect to $\kappa _t$.

A2 $$\begin{aligned} \frac{d}{{d\kappa _t}} \left( \nu \cdot \log (d_{xt}) \right) = 0 \end{aligned}$$

Thirdly, in the context of the COM–Poisson distribution, $Z(\lambda _{xt}, \nu )$ is a normalization constant called the partition function. It ensures that the probability mass function (PMF) of the COM–Poisson distribution sums up to 1 over all possible values of the random variable.

The exact form of $Z(\lambda _{xt}, \nu )$ is given by:

$$\begin{aligned}Z(\lambda _{xt}, \nu ) = \frac{{e^{\nu \lambda ^{1/\nu }}}}{{\lambda ^{(\nu -1)/(2\nu )} \cdot (2\pi )^{(\nu -1)/2} \cdot \sqrt{\nu }}}\end{aligned}$$

So that, differentiating $Z(\lambda _{xt}, \nu )$ we have:

$$\begin{aligned} & \frac{{d}}{{d\kappa _t}} \log Z(\lambda _{xt}, \nu ) = \\ & \quad \frac{{d}}{{d\kappa _t}}\left( \nu \cdot \lambda _{xt}^{1/\nu } - \left( \frac{{\nu -1}}{{2\nu }} \right) \cdot \log \lambda _{xt} - \frac{{\nu -1}}{{2}} \cdot \log (2\pi ) - \frac{{1}}{{2}} \cdot \log \nu \right) \end{aligned}$$

Now, with substituting $\lambda _{xt}$, let’s differentiate the four terms separately:

First term:

$$\begin{aligned} & \frac{{d}}{{d\kappa _t}} \left( \nu \cdot \left( L_{xt} \cdot e^{\alpha _x + \beta _x \cdot \kappa _t} \right) ^{1/\nu } \right) = \frac{{1}}{{\nu }} \cdot \nu \cdot \left( L_{xt} \cdot e^{\alpha _x + \beta _x \cdot \kappa _t} \right) ^{\frac{{1}}{{\nu }} - 1} \cdot L_{xt} \cdot \beta _x \cdot e^{\alpha _x + \beta _x \cdot \kappa _t} \\ & \quad \frac{{d}}{{d\kappa _t}} \left( \nu \cdot \left( L_{xt} \cdot e^{\alpha _x + \beta _x \cdot \kappa _t} \right) ^{1/\nu } \right) = \beta _x \cdot \left( L_{xt} \cdot e^{\alpha _x + \beta _x \cdot \kappa _t} \right) ^{\frac{{1}}{{\nu }} - 1} \cdot L_{xt} \cdot e^{\alpha _x + \beta _x \cdot \kappa _t}\end{aligned}$$

Second term:

$$\begin{aligned} & \frac{{d}}{{d\kappa _t}} \left( \left( \frac{{\nu -1}}{{2\nu }} \right) \cdot \log \left( L_{xt} \cdot e^{\alpha _x + \beta _x \cdot \kappa _t} \right) \right) = \left( \frac{{\nu -1}}{{2\nu }} \right) \cdot \frac{{1}}{{L_{xt} \cdot e^{\alpha _x + \beta _x \cdot \kappa _t}}} \cdot L_{xt} \cdot \beta _x \cdot e^{\alpha _x + \beta _x \cdot \kappa _t}\\ & \quad \frac{{d}}{{d\kappa _t}} \left( \left( \frac{{\nu -1}}{{2\nu }} \right) \cdot \log \left( L_{xt} \cdot e^{\alpha _x + \beta _x \cdot \kappa _t} \right) \right) = \frac{{\nu -1}}{{2\nu }} \cdot \beta _x\end{aligned}$$

Since the remaining (third and fourth) terms do not depend on $\kappa _t$, their derivatives are zero.

Combining the results, we have:

$$\begin{aligned} \frac{{d}}{{d\kappa _t}} \log Z(\lambda _{xt}, \nu ) = \beta _x \cdot \left( L_{xt} \cdot e^{\alpha _x + \beta _x \cdot \kappa _t} \right) ^{\frac{{1}}{{\nu }} - 1} \cdot L_{xt} \cdot e^{\alpha _x + \beta _x \cdot \kappa _t} - \frac{{\nu -1}}{{2\nu }} \cdot \beta _x\end{aligned}$$

Simplifying further, we get:

$$\begin{aligned} \frac{{d}}{{d\kappa _t}} \log Z(\lambda _{xt}, \nu ) = \beta _x \cdot \left( L_{xt} \cdot e^{\alpha _x + \beta _x \cdot \kappa _t} \right) ^{\frac{{\nu -1}}{{\nu }}} - \frac{{\nu -1}}{{2\nu }} \cdot \beta _x\end{aligned}$$

Substituting back $\lambda _{xt} = L_{xt} \cdot e^{\alpha _x + \beta _x \cdot \kappa _t}$, we have:

A3 $$\begin{aligned} & \frac{{d}}{{d\kappa _t}} \log Z(\lambda _{xt}, \nu ) = \beta _x \cdot \left( \lambda _{xt} \right) ^{\frac{{\nu -1}}{{\nu }}} - \frac{{\nu -1}}{{2\nu }} \cdot \beta _x\nonumber \\ & \quad \frac{{d}}{{d\kappa _t}} \log Z(\lambda _{xt}, \nu ) = \beta _x \cdot \left( \left( \lambda _{xt} \right) ^{\frac{{\nu -1}}{{\nu }}} - \frac{{\nu -1}}{{2\nu }} \right) \nonumber \\ & \quad \frac{{d}}{{d\kappa _t}} \log Z(\lambda _{xt}, \nu ) \approx \beta _x \cdot \left( \lambda _{xt} - \frac{{\nu -1}}{{2\nu }} \right) \end{aligned}$$

Therefore, combining Eqs. (A1–A3), the derivative of $\log P(X=d_{xt})$ with respect to $\kappa _t$ is approximately $\beta _x \cdot \left( d_{xt} - \lambda _{xt} + \frac{{\nu -1}}{{2\nu }} \right)$. So when the $\nu = 1$, the partial score is $\beta _x \cdot \left( d_{xt} - \lambda _{xt} \right)$

### A.2 Partial information matrix ($I_{xt}$)

Given $\lambda _{xt} = L_{xt} \cdot e^{\alpha _x + \beta _x \cdot \kappa _t}$, differentiation of the expression $\beta _x \cdot \left( d_{xt} - \lambda _{xt} + \frac{{\nu -1}}{{2\nu }} \right)$ with respect to $\kappa _t$ as below.

Using the chain rule, we have:

$$\begin{aligned} \frac{d}{d\kappa _t} \left( \beta _x \cdot \left( d_{xt} - \lambda _{xt} + \frac{{\nu -1}}{{2\nu }} \right) \right) = \beta _x \cdot \left( -\frac{d}{d\kappa _t} \lambda _{xt} \right) \end{aligned}$$

Now, we differentiate $\lambda _{xt}$ with respect to $\kappa _t$ using the chain rule:

$$\begin{aligned} & \frac{d}{d\kappa _t} \left( L_{xt} \cdot e^{\alpha _x + \beta _x \cdot \kappa _t} \right) = L_{xt} \cdot \frac{d}{d\kappa _t} \left( e^{\alpha _x + \beta _x \cdot \kappa _t} \right) \\ & \quad \frac{d}{d\kappa _t} \left( L_{xt} \cdot e^{\alpha _x + \beta _x \cdot \kappa _t} \right) = L_{xt} \cdot \beta _x \cdot e^{\alpha _x + \beta _x \cdot \kappa _t}\end{aligned}$$

Substituting this back into the expression:

$$\begin{aligned} \beta _x \cdot \left( -\beta _x \cdot L_{xt} \cdot e^{\alpha _x + \beta _x \cdot \kappa _t} \right) \end{aligned}$$

Simplifying further:

$$\begin{aligned}-\beta _x^2 \cdot L_{xt} \cdot e^{\alpha _x + \beta _x \cdot \kappa _t}\end{aligned}$$

Thus, the result for the partial information matrix ($I_{xt}$) is $\beta _x^2 \cdot L_{xt} \cdot e^{\alpha _x + \beta _x \cdot \kappa _t}$, or can be simplified by $\beta _x^2 \cdot \lambda _{xt}$.

## References

[CR1] Azman S, Pathmanathan D (2022) The GLM framework of the Lee–Carter model: a multi-country study. J Appl Stat 49(3):752–763 35706766 10.1080/02664763.2020.1833183 PMC9041986

[CR2] Blasques F, Koopman SJ, Lucas A(2014) Maximum likelihood estimation for generalized autoregressive score models (Tech. Rep.). Tinbergen Institute Discussion Paper No. 14-029/III. https://hdl.handle.net/10419/98908

[CR3] Böhnstedt M, Gampe J, Putter H (2021) Information measures and design issues in the study of mortality deceleration: findings for the gamma-Gompertz model. Lifetime Data Anal 27(3):333–356 33630224 10.1007/s10985-021-09518-4 PMC8238756

[CR4] Borges P, Rodrigues J, Balakrishnan N, Bazán J (2014) A COM–Poisson type generalization of the binomial distribution and its properties and applications. Stat Prob Lett 87:158–166

[CR5] Brouhns N, Denuit M, Vermunt JK (2002) A Poisson log-bilinear regression approach to the construction of projected lifetables. Insurance Math Econom 31(3):373–393

[CR6] Camarda CG, Basellini U (2021) Smoothing, decomposing and forecasting mortality rates. Eur J Popul 37:569–602 34421446 10.1007/s10680-021-09582-4 PMC8333270

[CR7] Chen H, MacMinn RD, Sun T (2017) Mortality dependence and longevity bond pricing: a dynamic factor copula mortality model with the GAS structure. J Risk Insurance 84(S1):393–415

[CR8] Cossette H, Delwarde A, Denuit M, Guillot F, Marceau É (2007) Pension plan valuation and mortality projection: a case study with mortality data. North Am Actuarial J 11(2):1–34

[CR9] Cox, D.R.(2006). Principles of statistical inference . Cambridge university press

[CR10] Creal D, Koopman SJ, Lucas A (2008) A general framework for observation driven time-varying parameter models. Tinbergen Institute Discussion Paper No. 08-108/4. 10.2139/ssrn.1297183

[CR11] Creal D, Koopman SJ, Lucas A (2011) A dynamic multivariate heavy-tailed model for time-varying volatilities and correlations. J Bus Econ Stat 29(4):552–563

[CR12] Creal D, Koopman SJ, Lucas A (2013) Generalized autoregressive score models with applications. J Appl Economet 28(5):777–795

[CR13] Crotty SM, Holland BR (2022) Comparing partitioned models to mixture models: Do information criteria apply? Syst Biol 71(6):1541–1548 35041002 10.1093/sysbio/syac003 PMC9558833

[CR14] Delwarde A, Denuit M, Eilers P (2007) Smoothing the lee-carter and poisson log-bilinear models for mortality forecasting: a penalized log-likelihood approach. Stat Model 7(1):29–48

[CR15] Dennis B, Ponciano JM, Taper ML, Lele SR (2019) Errors in statistical inference under model misspecification: evidence, hypothesis testing, and aic. Front Ecol Evol 7:372 34295904 10.3389/fevo.2019.00372 PMC8293863

[CR16] Francis RA, Geedipally SR, Guikema SD, Dhavala SS, Lord D, LaRocca S (2012) Characterizing the performance of the Conway–Maxwell poisson generalized linear model. Risk Anal An Int J 32(1):167–183 10.1111/j.1539-6924.2011.01659.x 21801191

[CR17] Gao G, Shi Y (2023) Robustness and spurious long memory: evidence from the generalized autoregressive score models. Ann Operat Res. 10.1007/s10479-023-05484-2

[CR18] Gaunt RE, Iyengar S, Olde Daalhuis AB, Simsek B (2019) An asymptotic expansion for the normalizing constant of the Conway–Maxwell–Poisson distribution. Ann Inst Stat Math 71:163–180

[CR19] Giacometti R, Bertocchi M, Rachev ST, Fabozzi FJ (2012) A comparison of the Lee–Carter model and ar–arch model for forecasting mortality rates. Insurance Math Econom 50(1):85–93

[CR20] Harvey, AC (2013) Dynamic models for volatility and heavy tails: with applications to financial and economic time series Vol: 52. Cambridge University Press

[CR21] Harvey AC, Chakravarty T (2008) Beta-t-(e) garch. Working Papers in Economics, Faculty of Economics, Cambridge University, UK.

[CR22] Head JD, Zerner MC (1985) A Broyden–Fletcher–Goldfarb–Shanno optimization procedure for molecular geometries. Chem Phys Lett 122(3):264–270

[CR23] Hilton J, Dodd E, Forster JJ, Smith PW (2019) Projecting UK mortality by using Bayesian generalized additive models. J R Stat Soc Ser C Appl Stat 68(1):29–49

[CR24] Hong WH, Yap JH, Selvachandran G, Thong PH, Son LH (2021) Forecasting mortality rates using hybrid lee-carter model, artificial neural network and random forest. Complex Intell Syst 7:163–189

[CR25] Jamal A, Mahmood T, Riaz M, Al-Ahmadi HM (2021) Glm-based flexible monitoring methods: an application to real-time highway safety surveillance. Symmetry 13(2):362

[CR26] Jdanov DA , Jasilionis D, Shkolnikov VM, Barbieri M (2019) Human mortality database. Encyclopedia of gerontology and population aging/editors Danan Gu, Matthew E. Dupre. Cham: Springer International Publishing, 2020

[CR27] Jørgensen B, Paes De Souza MC (1994) Fitting tweedie’s compound poisson model to insurance claims data. Scand Actuar J 1994(1):69–93

[CR28] Lee RD, Carter LR (1992) Modeling and forecasting us mortality. J Am Stat Assoc 87(419):659–671

[CR29] León Á, Rubio G, Serna G (2005) Autoregresive conditional volatility, skewness and kurtosis. Q Rev Econ Finance 45(4–5):599–618

[CR30] Li J (2013) A Poisson common factor model for projecting mortality and life expectancy jointly for females and males. Popul Stud 67(1):111–126. 10.1080/00324728.2012.689316 10.1080/00324728.2012.689316 22788919

[CR31] Li J, Pitt D, Li H (2022) Dispersion modelling of mortality for both sexes with Tweedie distributions. Scand Actuar J 2022(4):356–374

[CR32] Luengo D, Martino L, Bugallo M, Elvira V, Särkkä S (2020) A survey of monte Carlo methods for parameter estimation. EURASIP J Adv Signal Process 2020(1):1–62

[CR33] Mahmood T (2020) Generalized linear model based monitoring methods for high-yield processes. Qual Reliab Eng Int 36(5):1570–1591

[CR34] Mahmood T, Balakrishnan N, Xie M (2021) The generalized linear model-based exponentially weighted moving average and cumulative sum charts for the monitoring of high-quality processes. Appl Stoch Model Bus Ind 37(4):703–724

[CR35] Mahmood T, Sanusi RA, Xie M (2019) Flexible monitoring methods for high-yield processes. International workshop on intelligent statistical quality control (PP 45–63). In: Knoth, S., Schmid, W. (eds) Frontiers in Statistical Quality Control 13. ISQC 2019. Frontiers in Statistical Quality Control. Springer, Cham. 10.1007/978-3-030-67856-2_4

[CR36] Mammadova U, Özkale MR (2021) Profile monitoring for count data using poisson and Conway–Maxwell–Poisson regression-based control charts under multicollinearity problem. J Comput Appl Math 388:113275

[CR37] Mustafa F , Sherwani RAK, Raza MA (2023) A new exponentially weighted moving average control chart to monitor count data with applications in healthcare and manufacturing. J Stat Comput Simul 93(18):3308–3328

[CR38] Neves C, Fernandes C, Hoeltgebaum H (2017) Five different distributions for the lee-carter model of mortality forecasting: Aacomparison using gas models. Math Econ Insur, pp 7548–57

[CR39] Nigri A, Levantesi S, Marino M, Scognamiglio S, Perla F (2019) A deep learning integrated Lee–Carter model. Risks 7(1):33

[CR40] Peters GW, Shevchenko PV, Wüthrich MV (2009) Model uncertainty in claims reserving within Tweedie’s compound poisson models. ASTIN Bull J IAA 39(1):1–33

[CR41] Rabbi AMF, Mazzuco S (2021) Mortality forecasting with theLee–Carter method: adjusting for smoothing and lifespan disparity. Eur J Popul 37:97–120 33603592 10.1007/s10680-020-09559-9 PMC7865054

[CR42] Rakhmawan S.A, Omar M.H, Riaz M, Abbas N (2023) Hotelling T2 control chart for detecting changes in mortality models based on machine-learning decision tree. Mathematics 11(3):566

[CR43] Rasheed HA, Sadik NJ, Algamal ZY (2022) Jackknifed Liu-type estimator in the Conway–Maxwell poisson regression model. Int J Nonlinear Anal Appl 13(1):3153–3168

[CR44] Ratnasari V, Aviantholib IC, Dani ATR et al. (2022) Parameter estimation and hypothesis testing the second order of bivariate binary logistic regression (s-bblr) model with berndt hall-hall-hausman (bhhh) iterations. Commun Math Biol Neurosci 35:1–31

[CR45] Renshaw A.E, Haberman S (2006) A cohort-based extension to the Lee–Carter model for mortality reduction factors. Insurance Math Econom 38(3):556–570

[CR46] Renshaw A.E, Haberman S (2008) On simulation-based approaches to risk measurement in mortality with specific reference to poisson lee–carter modelling. Insurance Math Econom 42(2):797–816

[CR47] Schielzeth H, Dingemanse NJ, Nakagawa S, Westneat DF, Allegue H, Teplitsky C, Araya-Ajoy YG (2020) Robustness of linear mixed-effects models to violations of distributional assumptions. Methods Ecol Evol 11(9):1141–1152

[CR48] Sellers KF, Borle S, Shmueli G (2012) The Com–Poisson model for count data: a survey of methods and applications. Appl Stoch Model Bus Ind 28(2):104–116

[CR49] Shmueli G, Minka TP, Kadane JB, Borle S, Boatwright P (2005) A useful distribution for fitting discrete data: revival of the Conway–Maxwell–Poisson distribution. J R Stat Soc Ser C Appl Stat 54(1):127–142

[CR50] Taylor G (2007) Chain ladder for tweedie distributed claims data. Centre for Actuarial Studies, Department of Economics, University of Melbourne

[CR51] Zhao W (2021) A Broyden-Fletcher-Goldfarb-Shanno algorithm for reliability-based design optimization. Appl Math Model 92:447–465

[CR52] Zhou J, Li H, Zhong W (2021) A modified Diebold–Mariano test for equal forecast accuracy with clustered dependence. Econ Lett 207:110029

